# Supplementary material for: Prognosis of Advanced Heart Failure Patients according to Their Hemodynamic Profile Based on the Modified Forrester Classification
Source: J Clin Med. 2022 Jun 24;11(13):3663. doi: 10.3390/jcm11133663 (PMC9267518; doi:10.3390/jcm11133663)
Supplement: Supplementary file 1 [file jcm-11-03663-s001.zip › jcm-1753785-supplementary.pdf]

Supplemental Table S1. Adjusted association of Forrester's profiles with outcomes according to PCWP and CVP.

Model adjusted on age, gender and eGFR				
**According to CVP**	Warm-Dry	Cold-Dry	Warm-Wet	Cold-Wet
**Waitlist death or urgent heart transplant or LVAD implantation**	Reference	1.79 (0.46–6.93) p=0.399	0.88 (0.25–3.14) p=0.842	**3.40 (1.12–10.29) p=0.030**
**According to PCWP**	Warm-Dry	Cold-Dry	Warm-Wet	Cold-Wet
**Waitlist death or urgent heart transplant or LVAD implantation**	Reference	2.00 (0.53–7.57) p=0.308	1.25 (0.33–4.67) p=0.742	2.82 (0.86–9.24) p=0.087

CVP= central veinous pressure; eGFR = estimated glomerular filtration rate; LVAD = left ventricular assist device; PCWP= pulmonary capillary wedge pressure
